# Artificial Intelligence in Nephrology—State of the Art on Theoretical Background, Molecular Applications, and Clinical Interpretation

**DOI:** 10.3390/ijms27031285

**Published:** 2026-01-28

**Authors:** Jakub Stojanowski, Tomasz Gołębiowski, Kinga Musiał

**Affiliations:** 1 Department of Nephrology and Transplantation Medicine, Wroclaw Medical University, Borowska 213, 50-556 Wroclaw, Poland; jakub.stojanowski@student.umw.edu.pl (J.S.); tomasz.golebiowski@umw.edu.pl (T.G.); 2 Department of Pediatric Nephrology, Wroclaw Medical University, Borowska 213, 50-556 Wroclaw, Poland

**Keywords:** machine learning, deep learning, kidney, acute kidney injury, chronic kidney disease, metabolomics, proteomics, genomics

## Abstract

Artificial intelligence (AI) has transformed the clinical approach to analysis of large datasets, introducing the possibility of verifying long-term observations. AI tools ease the analysis of connections between multiple variable parameters and are particularly useful in the field of nephrology. These solutions enable the search for early diagnostic markers and predictors of renal function deterioration, both in acute and chronic conditions. Furthermore, AI techniques can be used as data mining tools, paving the way for future theories regarding the pathomechanisms of disease. Moreover, recently published papers focus on building models that facilitate decision-making, thus predicting renal involvement, its progression, and systemic complications. This review aims to demonstrate the multifunctionality of various AI methods from an omics perspective. To increase the power of argumentation, a mathematical background of each method is presented, followed by examples of molecular applications and anchorage in the nephrological clinical context. Our aim was to demonstrate the potential of AI tools in addressing diagnostic, prognostic, and therapeutic challenges, as well as to initiate the discussion on the pros and cons of future AI applications in nephrology.

## 1. Introduction

According to the founder of artificial intelligence, John McCarthy, AI relies on mimicking human decision-making [1]. From a clinical perspective, intelligence can mean predicting adverse outcomes in order to prevent them. In the era of big data, such results can only be achieved with advanced tools that enable in-depth analysis of a large number of variables, preferably assessed periodically during a long-term follow-up. International registries and multicenter studies, where the primary focus is on assessing risk factors associated with patient morbidity and mortality, serve as excellent examples. Clinical reasoning based on large-scale retrospective analysis approximates the most likely scenarios, identifies the most common endpoints, and provides solutions to prevent the disease, treat it effectively, or at least maintain in remission. The need for effective and early prediction of adverse events has dramatically increased during the COVID-19 pandemic.

There is a noticeable upward trend in interest in AI in medicine, as evidenced by bibliographic statistics. In the early 1990s, a single article per year was published on AI in medicine. Over the following years, the number of papers increased year after year, accelerating rapidly between 2017 and 2019, reaching several thousand papers annually [2]. This may be due to two leading factors: (1) advances in AI technology, and (2) the isolation caused by the COVID-19 pandemic and the pressure to conduct research under limited conditions and resources [3,4].

Global mortality risk management has fueled intensive research into the ability to effectively predict complications and improve decision-making. Nephrological aspects have also been of interest, including the prediction of acute kidney injury, morbidity and mortality in intensive care units, and thrombotic microangiopathies as the main nephrological complications of COVID-19 infection. Certain significant relationships also elude classical statistical analysis, and given this need, AI is prioritized for implementation.

Most diagnostic and therapeutic processes in nephrology utilize biochemical assays and are based on molecular aspects of the disease processes. Hence, there is significant interest in analyzing omics data, including proteomics and genomics.

Given the overwhelming abundance of data, rapid risk assessment may not directly translate to the needs of a specific patient. Experience and knowledge may not be sufficient in such cases, but with the help of AI, new diagnostic and therapeutic guidelines can be developed, based on a shift from macroscopic analysis to an individualized approach.

The potential for AI applications in nephrology is enormous, provided the method is tailored to specific requirements and endpoints. This review will discuss a wide range of AI tools, highlighting their importance in various clinical conditions and tailoring results to individual patient needs. It will also demonstrate, using examples from the literature, how to choose among many AI tools and which method suits best which disease.

## 2. General Classification

The most general classification of AI methods divides them into machine learning and deep learning. The first of these fields concerns rather simple methods, relatively less advanced than deep learning. The latter uses more powerful technical and algorithmic resources and provides a wider range of possible applications, although it is weak in certain areas which are reserved for machine learning.

### 2.1. Machine Learning

There are various machine learning techniques that are applicable to relevant topics. Some techniques require the supervision of a researcher, whose role is to determine the context of the data, i.e., assign them appropriate classes or labels. This type of AI that requires human intervention in the learning process is called supervised learning. In clinical practice, it may be useful to detect outlying structures and elements; then, the context is given by an algorithm. The mentioned technique is called unsupervised learning. However, when dynamic interaction of the algorithm with a changing environment is required, e.g., a changing patient’s condition, then reinforcement learning may be useful.

### 2.2. Unsupervised Learning

Unsupervised learning is based on finding patterns in data without given labels or target points. An example application is clustering and detecting deviations in a group of records or images, e.g., biopsy. Unsupervised learning methods allow for redefining existing diagnostic standards and reclassifying existing divisions by comparing them with existing classifications. Clustering, or cluster analysis, plays a special role in unsupervised machine learning. These techniques involve grouping elements based on similarity.

Consequently, they empower identification of parameters or constellations of parameters responsible for belonging to a specific cluster, also known as a category. This approach allows for reclassification of chronic kidney disease stages based on the results of analysis using the Self-Organizing Maps—an unsupervised ANN machine learning algorithm [5]. This approach has allowed the discovery of protein patterns, as well as sets of patterns characterizing CKD with an etiology confirmed by nephropathological examination.

A clustered dataset of prediabetic patients was analyzed using lasso regression. The stepwise application of several analysis techniques allowed for the stratification of patient groups based on selected peptidomes and the determination of the risk of developing diabetes and diabetic complications [6].

In other applications, unsupervised learning allows for the detection of important elements in images, e.g., examination of histopathological kidney preparations, and linking them with the patient’s laboratory parameters [7]. Modeling using unsupervised machine learning allows for stratification of the risk of transplant rejection and the risk of death in the observed period [8]. Patient clustering is possible with a clear distinction between patient group characteristics and graft survival in transplant patients [9].

### 2.3. Supervised Learning

Supervised learning requires input data that has been grouped into classes. This method typically involves labeling and categorizing data records. For example, a set of genes can be assigned to the degree of renal fibrosis, thus assessing the risk of kidney disease progression in a carrier of these genes. The use of several models in a parallel decision-making process allows to achieve the target point with an AUC of 0.923 based on the selected five genes: ARID4B, EOMES, KCNJ3 (alias GIRK-1), LIF, and STAT1 [10]. Looking back, hypotheses can be constructed based on the association of the endpoint with the designated genes, laying the foundation for further research to uncover the association of fibrosis with the expression of these genes.

Similarly, renal failure can be assessed based on the proteomic set detected in the patient’s urine using clustering [11]. Patients with CKD constitute a group with an interdisciplinary profile and complex outcomes. Medical history of CKD demonstrates a higher risk of cardiovascular complications, which can be predicted using a supervised machine learning model [12]. An example of molecular application is a database of patient records with parameters and the endpoint of achieving remission. In detail, the use of machine learning allows for the assessment of the risk of AKI in pediatric patients and adults staying in the ICU more effectively than traditional methods [13,14,15].

### 2.4. Reinforcement Learning

Reinforcement learning is like playing a game in which the player performs certain tasks in the game environment and receives feedback in the form of a reward and information about the game state. In a hospital setting, this mimics personalized medicine. While therapy is dynamically tailored to the patient’s condition, it is subject to subsequent adaptive changes. These changes involve not only drug dosages but also the introduction of new medications, the discontinuation of old ones, and the expansion of diagnostics. An example application is the personalized dosing of the sedatives (propofol and fentanyl) in response to variable patient characteristics [16]. Therapy modification, based on reinforcement learning, has been used to achieve optimal dry weight in patients undergoing hemodialysis [17]. Likewise, the above-mentioned AI method allowed the modulation of erythropoiesis-stimulating agent (ESA) dosing in relation to variable patient characteristics and outcomes [18,19].

## 3. Machine Learning Models

### 3.1. Random Trees

Random forest classifiers (RFCs) are derived from classification and regression trees (CARTs), which are models based on decision trees built using a splitting procedure. The result of such procedures is a decision tree, which, due to the algorithm and method of generation, is highly sensitive to variability in input parameters. Random forest classifiers allow for improved flexibility of the decision tree by utilizing multiple decision trees built on subsamples of the input dataset.

A specific example of decision trees are guideline-based algorithms. The essence of a decision tree is to apportion an input set using successive conditions, based on a minority relation, in the simplest possible way. The goal of a decision tree is to divide the set in such a way that one class is separated from another in the optimal number of steps. RFCs are insensitive to data that may be irrelevant. In practice, this means that the random forest’s construction method forces the ignoring of variables that do not provide crucial information for partitioning the dataset. An RFC classifier consists of independently generated decision trees. Therefore, RFCs are characterized by a certain degree of robustness to outliers.

Compared to other methods selected by Yuan et al., an RFC based on selected genes allows for the extraction of a dataset most helpful in analyzing the risk of renal fibrosis, which other machine learning techniques assess [10]. The role of the classifier can be played by data mining to find the most valuable input datasets. To this end, the small or large deterioration in prediction occurs after eliminating a variable. If such a variable has little impact on the classifier’s result, it is likely not significant in the prediction and can be eliminated.

The RFC has been used to assess potential drug interactions, achieving an accuracy of 96.51%. Competing models by Kha et al. achieved slightly better results with similar input data, but RFC offers several advantages, including lower resource requirements and faster evaluation [20].

RFCs, due to their simplicity, transparency, and short computational time, are used to discover features associated with mortality and risk factors for the development of diabetic kidney disease. An RFC-based model based on cystatin C, serum albumin, hemoglobin, 24-h total urinary protein, and eGFR predicted ESRD in patients with diabetic kidney disease with an accuracy of 82.65% and an AUC of 0.90 [21]. An RFC-based model based on diabetic retinopathy status, diabetes course, pulse pressure, hemoglobin level, serum creatinine level, albumin level, serum total cholesterol, and acute onset of severe proteinuria was able to distinguish the etiology of kidney disease between diabetic and nondiabetic kidney disease. In this case, adding variables slightly improved the predictive value [22].

### 3.2. Variants of Regression

Logistic regression is one of the basic techniques for analyzing dichotomous variables using odds analysis. The formal approach to logistic regression is a general linear model, where the link function is the logarithm of the odds of selecting a given category. Thus, logistic regression represents a very simple yet effective machine learning model. Machine learning analysis allows for the verification of known metabolic pathways, but it also generates a tool particularly useful in practical clinical applications. Knowing the developmental scenario of diabetic kidney disease and progression to subsequent stages based on logistic regression allows for the identification of a specific proteomic trace and the development of a tool for predicting patients at particularly high risk of disease progression [23].

Machine learning is widely used in data mining to identify key data. For this purpose, multiple models can be built, their performance assessed, and the best model identified. Decision-making parameters can be reviewed to draw clinical conclusions or suggestions for further research, including differentiation of nephropathies leading to chronic kidney disease [24].

LASSO regression (least absolute shrinkage and selection operator) is an extension of logistic regression in which regularization clarifies the leading problem and improves overall performance while reducing the risk of overfitting. Using RFC, Yuan et al. identified basement membrane gene markers associated with a leading mechanism of chronic kidney disease progression, namely renal parenchymal fibrosis. Based on parameters selected using three machine learning methods, a nomogram based on LASSO regression was constructed with satisfactory discriminative power, with an AUC of 0.923, enabling estimation of the risk of kidney disease progression.

A pre-clustered dataset of prediabetic patients, then analyzed with LASSO regression, allows for the stratification of patient groups based on selected peptidomes and the determination of the risk of developing diabetes and diabetic complications. Positive results for the CKD273 (CKD proteinogram classifier), HF2 (urine peptidogram to determine the risk of heart failure), and CAD238 (coronary artery disease proteomic classifier) tests were associated with increased insulin resistance. These parameters differentiated groups with a high risk of progression from groups with a very low and low risk of progression to diabetes and the development of diabetic complications [6].

The next evolution of logistic regression, and especially LASSO regression, is Elastic Net Regression with additional regularization; in short, a tool that improves predictions and reduces overfitting. Proteomic modeling and machine learning identify new risk management models and analyze the biological pathways of various proteins involved in the development of cardiovascular complications in patients with comorbidities and chronic kidney disease [12].

Regularized Cox Regression is a compilation of Elastic Net with the Cox hazard model. Combining selected techniques allows for the utilization of their advantages and mixing the partial results into a final solution to the overall problem. Massy et al. used clustering to determine CKD severity categories [11]. The number of peptides constituting the protein profile was narrowed using Regularized Cox Regression. Clustering allows for the identification of closely related data subpopulations and then the selection of data from which these populations can be successively differentiated. This technique is an extension of the Cox risk model, with a greater emphasis on avoiding overfitting [25].

### 3.3. eXtreme Gradient Boosting (XGBoost)

eXtreme Gradient Boosting (XGBoost) is a machine learning model containing decision trees based on the principle of boosting, i.e., enriching the existing model with a new additional model, provided it improves performance. The added models are decision trees that cover the errors of the existing model. XGBoost model is promising due to its good convergence to optimal performance and relatively low complexity. It has been used, among others, in diagnosing CKD, hyperkalemia in patients with CKD, or mortality in patients with acute kidney injury, with accuracy of 1.00, 0.933, 0.86, respectively [26,27,28]. XGBoost achieved the prediction of 50% reduction of estimated glomerular filtration rate (eGFR) and progression to end-stage kidney disease (ESKD) in patients with IgA nephropathy with C-statistic of 0.84 [29].

### 3.4. Support Vector Machine

Support vector machine (SVM) is a method that involves determining a hyperplane that optimally separates the elements of the set according to the assigned classes. This is a relatively simple method, but when used on a small amount of data in the form of a table, it may prove to be superior to RFC or XGBoost [30]. Proteomic analysis of potential factors with SVM associated with cellular aging may provide insight into the protein-mediated pathways responsible for the development of chronic kidney disease in patients without underlying disease compared to those with underlying disease but without established kidney disease. This sheds light on the independence of the renal disease process and provides a foundation for targeted medicine in nephrology [31].

### 3.5. K-Nearest Neighbors Classifier (kNN)

This technique relies on the similarity of an element to its nearest N neighbors, e.g., three. Based on the labels of the closest elements, the most frequently occurring one is selected and the similarity of a given element of the set to these labels is estimated.

The kNN classifier allows for group classification and clustering, in particular, effective differentiation of a group of patients with diabetic nephropathy and glomerulonephritis from a healthy control based on serum proteomics. Results had an accuracy of 96.3% for glomerulonephritris and 96.4% diabetic nephropathy [32]. Among other machine learning techniques, kNN also performed very well in kidney cell typing based on scRNA-seq and snRNA-seq data [33]. Models based on XGB, RFC, KNN, and SVM effectively identified blood cell types with a sufficiently high F1-score, varying depending on the model configurations, reaching values of 0.97–1.0. Identifying kidney cells proved more challenging. Here, F1-scores for nephron cell typing were significantly poorer, bordering on 0.6, and for proximal tubule cell identification, even values of 0.32–0.35. All the described models were based on input data consisting of scRNA and snRNA sequences obtained from kidney biopsies.

Compared to other machine learning techniques, kNN is suitable for data with a low number of dimensions, i.e., the number of features. Depending on the input data and selected variables, its performance is comparable to logistic regression, RFCs, or support vector machines [24,34]. It is clearly dominant in clustering due to the nature of the algorithm. However, an unusual application is data imputing, i.e., filling in missing data based on neighborhood similarity [35]. Data completeness is crucial for machine learning. Empty cells are interpreted as zero values or must be removed from columns and rows containing entire records, which reduces the size of the input data. Inputting the most likely values in empty cells improves model flexibility and performance [36].

## 4. Deep Learning and Multilayer Perceptron

Deep learning refers to AI techniques that are more advanced than machine learning and are based on convolutional neural networks. A convolutional neural network (CNN), like a multilayer perceptron, is a feedforward network, i.e., a one-way network in which information flows from input to output without returning to visited neurons or creating a reinforcement loop. Convolution means the optimization of neural networks with the use of mathematical operations based on the application of filters. Applying a filter ensures that subsequent layers gain some context about the input data. This application allows, for example, image recognition, finding details in the image, e.g., recognizing glomeruli in biopsies [37]. The anatomical analogy of a convolutional network is the visual cortex of the brain, where different neurons correspond to a specific visual field. Comprehensive CNN-based systems allow for the classification of biopsy specimens from rejected renal allografts in terms of the presence of pathology, and they also visually highlight pathological areas [38]. Deep learning allows for the differentiation of individual structures in histopathological images. Segmenting the histopathological image allows for automatic qualitative classification of the collected specimen into many groups, such as unilateral ureteral obstruction, ischemia-reperfusion injury, nephrotoxic serum nephritis, adenine-induced nephropathy, or Alport syndrome [39].

A niche, although essential, application of deep learning is in omics analysis. Its significance results from the ability to identify, e.g., the molecular profile of patients with advanced diabetic kidney disease, based on two lipid metabolites that have significant relationships with the level of glycated hemoglobin and glycemic profiles in patients with type 2 diabetes [40].

### 4.1. From Linear Regression to Artificial Neural Network

The main goal of statistical modeling is to describe a phenomenon using mathematical operations, with the smallest possible error between observed and calculated values. One of the simplest examples is linear regression, which assumes a linear relationship between the explained and explanatory variables. Assuming that the regression coefficients are betas (*β*) and the random error is epsilon (*ε*), then linear regression is a linear function that can be written as

$$y_{i}=\beta_{0}+\beta_{1}x_{1}+\beta_{2}x_{2}+\cdots +\beta_{k}x_{k}+\varepsilon_{i}$$

To determine the above coefficients, the least squares method and its derivatives are used. The purpose of such calculations is to minimize differences in estimates from observed values. The main issue then becomes how to calculate the coefficients to minimize the loss function describing the discrepancy between observations and approximations.

A single artificial neuron is called a perceptron and can be described as a composite of two functions: the activation function and the sum of the products of inputs *x*_1_, *x*_2_, *x*_3_, …, *x_i_* and their corresponding weights marked by *w*_1_, *w*_2_, *w*_3_, …, *w_i_* plus an intercept called bias vector *w*_0_, which is the argument of the activation function.

$$output=f\left(w_{0}+\sum_{i=1}^{k}w_{i}x_{i}\;\right)$$

Sum of products of inputs and weights assigned to inputs:

$$\sum_{i=1}^{k}w_{i}x_{i}=w_{1}x_{1}+w_{2}x_{2}+\cdots +w_{k}x_{k}$$

Activation functions can be various and may influence the behavior of the network differently, which is an empirical domain.

The simplest is rectifier or rectified linear unit (ReLU), which returns the positive part of the argument:

$$f\left(x\right)=max(0,x)$$

The use of the sigmoid function reduces a single neuron to the level of logistic regression:

$$f\left(x\right)=\frac{1}{1+e^{-x}}$$

Yet another activation function is the hyperbolic tangent function:

$$f\left(x\right)=\tanh\left(x\right)=\frac{e^{x}-e^{-x}}{e^{x}+e^{-x}}$$

**Figure 1:**
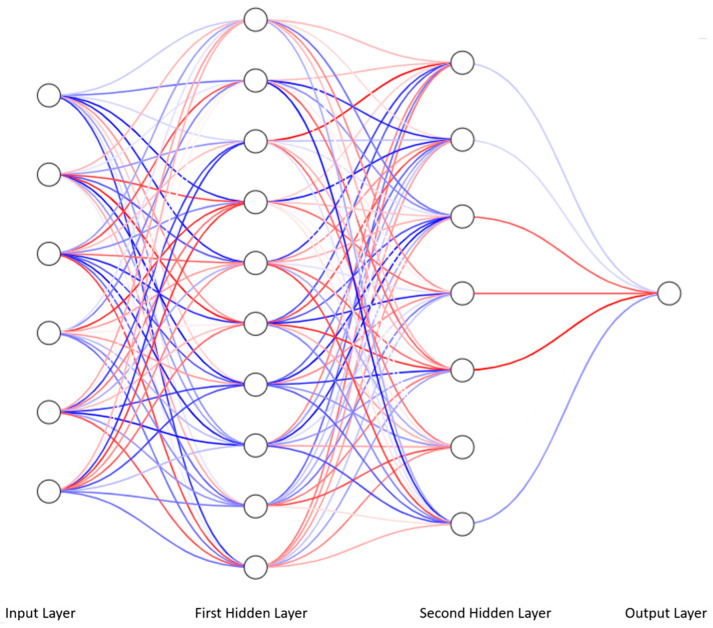
Fully connected neural network with an input layer containing 6 neurons, two hidden layers containing 10 and 7 neurons, respectively, and an output neuron constituting the output layer. The neurons are represented by white circles with black edges. The red color shows the positive weight value, and the blue color shows the negative weight value of the connection between the appropriate neurons. The color intensity is proportional to weight. Each neuron in one layer has connections with every neuron in the next layer. The visualizations were made using online software from the following website: https://alexlenail.me/NN-SVG/index.html. Accessed on 5 January 2026.

By arranging many neurons into layers and connecting the outputs of others with the inputs of others in an organized and orderly way, the resulting neural network is obtained (Figure 1).

If such a network has only perceptrons, i.e., neurons described above, which are organized into layers in which the neurons are connected each with each other between the layers but not within them, we obtain a model of an ANN called a multilayer perceptron (MLP). A multilayer perceptron has an input layer, optional hidden layers, and an output layer. Each neuron in one layer is connected to each neuron in the other layer, creating a complete neural network.

The multilayer perceptron-based prediction model was able to effectively predict fibrosis in patients with chronic kidney disease (CKD) based on leading predictors SWE value, eGFR, age, UACR, RI, renal parenchyma thickness and hypertension, and several with less impact on prediction [41]. On the other hand, a model based on static and dynamic parameters allowed for ongoing monitoring of the risk of intradialytic hypotension in hemodialysis patients, which is, in a sense, an example of reinforcement learning [42]. The mentioned model based on static features such as age, diabetes status, hypertension and ultrafiltration, and dynamic parameters combined with the mentioned static ones, heart rate (HR) slope at 0, 30, and 60 min for each patient and depending on nervous tension. At the same time, the authors used extended dynamic variables derived from HR slope at appropriate moments of measurement, difference of slope (DoS) at 30–15 min and 60–30 min. The model designed in this way achieved an accuracy of 81.5%. In the model, diabetes, age more than 65 years old, and a negative history of hypertension created a cluster that brought patients closer to the endpoint, i.e., hypotension.

Attempts are being made to reclassify chronic kidney disease, based on the results of analysis using the Self-Organizing Maps unsupervised machine learning algorithm [2]. This approach has allowed the discovery of protein patterns, as well as sets of patterns, characterizing CKD with an etiology confirmed by nephropathological examination. A Self-Organizing Map is a type of neural network that contains a two-dimensional array of data in the input layer, which is connected to several neurons in the competitive layer. Case analysis allows to formulate hypotheses based on the aforementioned modeling approach.

### 4.2. Deep Learning and Convolutional Neural Networks

**Figure 2:**
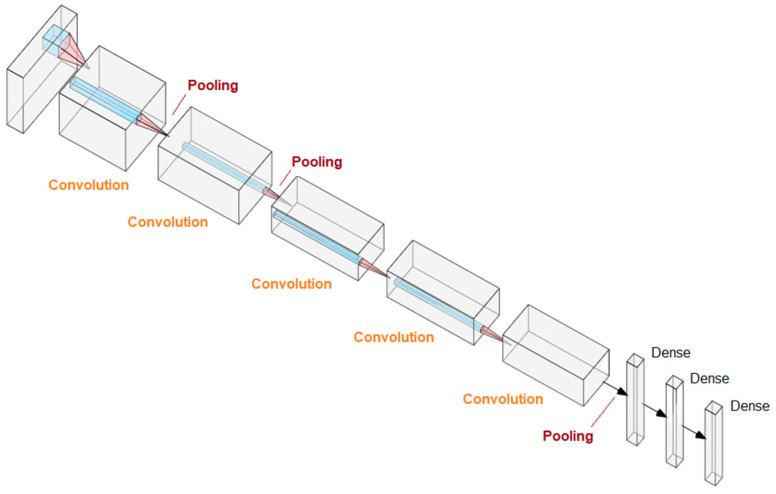
Visualization of a convolutional neural network on the example of the AlexNet architecture. The input image is processed in such a way that convolutional layers formulate the context and build features from the elements of the previous layer, and pooling layers combine these features, giving them a broader context. Ultimately, the set and arrangement of features allow for classifying the image, finding structures, segmenting or detecting deviations from the norm. The visualizations were made using online software from the following website: https://alexlenail.me/NN-SVG/index.html. Accessed on 5 January 2026.

Deep learning is based on the use of many layers of a neural network. A convolutional neural network uses convolution operations to determine the context of the analyzed data. Deep learning is used in analyzing radiological and histopathological images, including detecting differences in unsupervised learning or detecting specific structures or pathologies in supervised learning [37,38,39,43]. For example, the AlexNet architecture allowed segmenting the glomeruli in the histopathological image [43,44]. In detail, convolutional networks consist of three types of layers arranged appropriately: (1) convolutional layers that enable the creation of features from single data, e.g., pixels in an image, (2) pooling layers that are responsible for combining features and deepen the neural network, and (3) dense layers, which are responsible for classification (Figure 2).

Classification of histopathological images of kidney specimens using a network with U-net architecture allows for the identification of structures visible in the image in an automatic and precise way [37,45,46,47]. Networks from the ResNet family (ResNet18, 50 and 101), where the numbers indicate the depth of the network, or the number of layers, allow the classification of kidney transplant pathology [38,48].

The summary of multiple machine learning applications in translation of proteomics to clinical practice is given below (Table 1).

## 5. Comparative Characteristics of Various Methods

All classification models gain power as the volume of analyzed data increases and the structure of the classifier is modified. The role of the model is to match the provided data, but redundant data may obscure the classification process. Each model tries to adapt as much as possible to the provided data to ensure optimal performance. The role of a physician is to avoid unnecessary data by deciding which variables are of clinical significance and should be included within the dataset. Consequently, balancing the number of variables should have a positive impact on the classifier’s performance.

RFC has some ability to reject irrelevant variables, although this feature has a natural limitation, because the model tries to fit the training data anyway.

There is no scaling or normalization required to prepare the data before use in the RFC. The smallest cell in the decision tree in the RFC is the node with the minority relationship stored. In the case of a neural network, data scaling is necessary for the network to function properly. On the other hand, artificial neural networks are more amenable to incremental learning, which involves introducing new data into the model. RFCs require virtually complete redesign to expand the training input data.

Both mentioned-above models allow the detection of linear and non-linear dependencies, with non-linear performance being a definite advantage.

The complexity of the neural network may both bring a profit and expose to harm. Better fitting to non-linear data is advantageous, whereas complexity and difficulty in interpretation are inconvenient. Unlike decision trees in an RFC, where the stages of decision-making can be traced, a neural network involves many calculations, the meaning of which is not clear without careful analysis. To maintain transparency of the processes taking place inside the model, it is worth focusing on models that are simpler than neural networks, e.g., RFCs.

Fernández-Delgado et al. compared various machine learning models on the publicly available UCI Repository training dataset [49]. The conclusion was reached that as the data complexity increases, the accuracy of models based on neural networks increases, which confirms the validity of using neural networks in large and complex datasets. RFCs on the UCI repository datasets showed the highest accuracy, although neural networks were not significantly worse. These findings further reinforce the need to adapt AI methods to the quality of available data.

Classifiers based on decision trees, including RFCs or XGBoost, are less time-consuming and resource-intensive. RFC is partially insensitive to irrelevant variables and requires neither scaling nor standardization. Decision trees perform better on small amounts of data compared to neural networks. A big advantage is the transparency of the model, in which the decision-making process in individual trees can be traced.

The RFC algorithm allows for the extraction of data relevant to analysis. Input data selection is performed during model development. The extracted data provides the basis for further research. Similarly, significant factors influencing acute rejection of transplanted organs can be assessed, identifying individuals at particular risk before the procedure. This approach also allows for the estimation of the possibility of delayed function. In summary, transplantology is not only about modeling but also about data selection. The random forest classifier consisting of donor BMI, recipient BMI, donor-recipient weight difference, and donor eGFR before collection, EPTS, KDRI, KDPI, recipient gender, and age was very effective in identifying the risk of delayed graft function with an AUC of 0.91 and an accuracy of 93.75% [50].

Similarly, Liu et al. successfully identified the most important predictors of progression from IgA nephropathy to end-stage renal disease. Using random forests, sensitive data was extracted and a predictive model was built based on it. The model based on clinical data and kidney biopsy results allowed for predicting end-stage renal disease in patients diagnosed with IgA nephropathy [51]. A similar analysis methodology based on the comparison of machine learning techniques was performed by Konieczny et al. to predict remission of IgA nephropathy [52].

ANN has a complex structure capable of discovering non-linear relationships, but requires a sufficiently large amount of data to learn. ANN requires data rescaling and the performance improves after data standardization. Compared to an RFC, a neural network such as a multilayer perceptron requires the adjustment of more parameters, e.g., the number of neurons in hidden layers.

More complex neural networks such as deep learning are used in analyzing complex structures, finding patterns, or detecting irregularities. Additionally, deep learning may use data augmentation to improve the quality of classification. Deep neural networks allow for the most advanced data analysis of the presented methods. However, the greatest technical resources and a sufficiently large amount of good-quality data are necessary.

Table 2 summarizes major applications of AI methods, characterizing their requirements and major features.

## 6. Limitations

### 6.1. Missing Data

Databases, especially those collected over a long period of time, may have missing data due to individual cells or blocks. There may be missing data in individual patient records, or in the measurements corresponding to column variables. Artificial intelligence allows to solve the problem of missing data with the technical improvement of the algorithm’s performance, but it also distorts real observations. Methods such as random forest are insensitive to irrelevant variables and can independently eliminate their involvement in modeling. However, care should be taken to ensure that the results obtained are primarily related to the clinical situation, and not merely correct.

Missing data can be filled with, among others, some version of random forests, as well as kNN imputer [35]. The kNN method involves deciding based on the local surroundings of the point. Empty cells may be interpreted as a zero value depending on the computer environment and software. While creating a database, one should clearly distinguish between an empty cell and a parameter with a value of zero.

### 6.2. Low Number of Patient Records

The main aspect that limits the use of machine learning with a small dataset is the difficulty in training a model that always converges mathematically to the optimal one. It is difficult to cross-validate such a model because the divided subsets are very small and, at the same time, single errors significantly worsen the final result. However, this difficulty does not exclude the potential of machine learning efficiency on small sets. In the literature, it is possible to encounter the use of a RFC in genomics, where the data is often small and contains many variable parameters [34,49,50]. Our own experience has shown the possibility of using RFC in the prediction of kidney dysfunction among children after hematopoietic stem cell transplantation and the need for dialysis in those with chronic kidney disease [54,55].

An additional problem, affecting small datasets, is the difficulty in validation. Whereas large datasets provide reasonable division into a training set, in small datasets, such division carries the risk of distorting the measured results. A sufficiently large training set ensures adequate representation of individual classes in cross-validation and reliability of the measured results. Small sets raise the risk of individual element prevalence and significant disturbance in the measured performance. In the case of small datasets, leave-one-out cross-validation (LOOCV) can be used [56]. LOOCV involves cross-validation with each element validating the remaining elements of the training set, so the number of obtained measurements corresponds to the number of elements in the set. For large sets of elements, 5 or 10, cross-validation is performed, which similarly creates 5 or 10 divisions into the training and validation sets [14].

The intuitive hypothesis that a larger dataset improves prediction and classification performance is confirmed by experiments [57]. Certain issues, however, reach the limit of further refinement after a certain number of samples is reached [58]. This means that collecting more additional data, which is labor-intensive and time-consuming, may not yield a sufficiently large improvement in model performance. A significant change in the amount of data does not significantly impact the final result. A small number of samples can lead to overfitting to the training data and poor performance on the testing data, resulting in poor model performance. Based on the literature, it appears that an appropriate dataset size range would be 10 to 100 times the number of features in the dataset [57,58,59,60,61]. Naturally, this is a rough estimate and, especially in proteomics, may not be applicable when analyzing many proteins and protein products.

### 6.3. Large Number of Variable Parameters

A large number of parameters with a relatively small number of patient records may lead to overfitting the model to the training data and, as a result, show poor performance on new data. The risk of overfitting also applies to the models that are too complex. More advanced models, such as neural networks, have a complexity that allows complete adaptation to the training data. However, such a model may not be able to operate effectively on new data that is outstandingly different from the training data.

While designing the database and generating predictive models, the appropriateness of selecting specific features should be considered. The clinical justification of chosen numbers is essential, because only the researcher is aware of the context of the study and the clinical environment.

RFC allows to measure the impact of variables on the decision-making process with the Gini importance index, quantifying the contribution of a given variable to classification or regression. In this way, the number of input variables can be reduced by discarding the least important ones. New parameters relevant to the clinical problem under study may be discovered during the development of the model [53,62,63].

### 6.4. Selection of the Method

As demonstrated by Konieczny et al., various methods may achieve different results with the same input data, owing to the algorithm that was chosen [52]. There are no unanimous recommendations regarding the choice of a specific method or the decisive advantage of one technique over another. Certain methods, as summarized in Table 2, offer advantages inherent to their design. These include complexity, adaptability, robustness to large datasets, flexibility, and insensitivity to missing or bad data. Method selection is largely a case study of the clinical problem and the question posed [64].

### 6.5. Dependent Variables and Augmented Data

Classical statistical analysis struggles with the dependence of variables. In the case of machine learning, augmented data can be introduced. Augmented data is used in deep learning and example operations involve scaling or rotating the analyzed images [65]. In the case of tabular data analysis, data expansion may consist of introducing variables that are derivatives of other variables. BMI as a derivative of weight and height could serve as an example. The introduction of augmented data with a valid clinical or real-world context should be considered, as it provides context assigned by the researcher. A simple example is a classifier that assigns points within a circle with a given radius. The introduction of derived variables—coordinate squares—significantly simplifies the task. It should be noted that the time and technical complexity of models increase with the number of variables, but imposing variables is an expression of the researcher’s control over the model [66].

## 7. Accuracy Assessment Methods in Machine Learning

Various machine learning techniques are characterized by differential performance in relation to the input data. The main requirement is the completeness of the data used to generate the model. Moreover, capabilities of a model usually increase with its complexity, although a simpler model may perform better under certain conditions. Simpler models show some insensitivity to missing data or outlier data. RFCs do not require either scaling or standardization of data, whereas deep networks use scaling, standardization, and augmentation of data to improve performance. Although greater computational complexity is the consequence, the significantly improved performance may compensate for it. Models based on decision trees are clearer in interpretation than neural networks, yet the neural networks provide more possibilities and potential applications. However, due to their complexity and extensive structure, the manual interpretation is difficult or practically impossible.

Below is a confusion matrix defined in a general sense:

$$M=\left[\begin{array}{cc} TP & FN\\ FP & TN \end{array}\right]$$

Correctly classified values are *TP* (true positive) and *TN* (true negative), respectively, and incorrectly classified values are *FP* (false positive) and *FN* (false negative), respectively. An ideal classifier is the one that correctly distinguishes true and false instances, and therefore, the *FP* and *FN* values under such conditions are zero.

Based on such a matrix, various measures of model behavior can be derived. However, it should be taken into account whether the dataset is balanced in terms of the presence of both positive and negative instances.

The Matthews correlation coefficient (*MCC*) is a binary classifier metric that is based on all fields of the confusion matrix and is particularly useful in unbalanced sets. Additionally, this metric is not oriented towards the positive class and remains symmetrical towards the negative class [67,68].

$$MCC=\frac{TP\cdot TN-FP\cdot FN}{\sqrt{\left(TP+FP\right)\cdot \left(TP+FN\right)\cdot \left(TN+FP\right)\cdot \left(TN+FN\right)}}$$

In our previous work, we have emphasized high *MCC* scores of our models [69]. *MCC* takes values in the range from −1.0 to 1.0

Accuracy is the most intuitive parameter determining the ratio of correct classifications to all instances.

$$Accuracy=\frac{TP+TN}{All\;the\;instances}=\frac{TP+TN}{TP+FP+FN+TN}$$

This parameter is sensitive to the balance of the set. For example, if the set contains 90 data of one class and 10 of the other class, the classifier assigning the dominant class to any sample will obtain *TP* 90, *FP* 10, *FN* 0, TN 0, which gives an accuracy of 0.9.

*Precision*, also called positive prediction value, is the probability that a positive result is true.

$$Precision=\frac{TP}{TP+FP}$$

*Recall*, also called sensitivity, is the probability of detecting a positive instance.

$$Recall=\frac{TP}{TP+FN}$$

The receiver-operator curve (ROC) shows how the true positive rate (TPR) changes in relation to the false positive rate (FPR) and is a commonly used metric describing the discriminatory ability of a binary classifier. The area under the ROC (AUROC) determines how close the measured classifier is to the ideal classifier (AUROC = 1.0).

*F*_1_*-score* is the harmonic mean of precision and recall, a simplified measure relative to MCC, but not taking into account true negative classifications.

$$F_{1}score=\frac{2}{{precision}^{-1}+{recall}^{-1}}=\frac{2\cdot precision\cdot recall}{precision+recall}=\frac{2TP}{2TP+FP+FN}$$

## 8. How to Choose an Appropriate AI Tool? Practical Summary

Research in the field of nephrology is often conducted on small groups of patients because it often involves disorders that are rare in the general population. The utilization of standard statistical techniques to analyze research outcomes on small sample sizes may result in the inability to achieve statistical significance. Additionally, missing data, outlier data, or a large number of variables may further complicate the interpretation of results.

In such conditions, AI techniques can be extremely helpful in prediction of development, risk factors, mortality in a variety of diseases, including glomerulonephritis, AKI, CKD, and indications for renal replacement therapy. The range of applications varies from imaging diagnostics, through surgery and omics, to genetic testing.

RFCs do not require either scaling or standardization of data, whereas deep networks use scaling, standardization, and augmentation of data to improve performance. Models based on decision trees are clearer in interpretation than neural networks, whereas the neural network provides more possibilities and potential applications. However, due to its complexity and extensive structure, manual interpretation is difficult.

Therefore, the appropriate selection of data for analysis is essential for machine learning. Missing data clouds the picture, so removing such records or columns and not including them in the analysis should be considered. However, such an attitude may become problematic in the case of rare diseases. Missing data imputation using the kNN method may be the best solution, as it can to some extent deduce what the missing cell may look like, based on the neighborhood similarity.

Regardless of the chosen technique, it is essential to assess the effectiveness of any model, based on the accuracy assessment, mainly the Matthews correlation coefficient and AUROC, as well as on the external validation.

## 9. AI-Driven Proteomic Diagnostics—Recent Nephrological Perspective

Recent years in medicine have been marked by extensive AI-driven research in the field of nephrology.

Among multiple trigger factors, the COVID-19 pandemic seemed the most stimulating one, paving the way for analysis with the use of artificial intelligence tools. Based on the known pathways of relationships between inflammatory markers, cytokines, and chemokines, one could specifically ask artificial intelligence what conclusions can be drawn using algorithms more complex than the classical analysis [70]. The virus was detected in kidney cells, and the pathogen itself, through metabolic and proteomic pathways, triggered a cytokine storm, damaging almost every organ and system in the body [71,72,73]. The Polish CRACoV-AKI prognostic model enabled the stratification of patients with SARS-CoV-2 infection, according to the risk of development of acute kidney injury [74]. Therefore, the SARS-CoV-2 pandemic has fueled medicine based on molecular diagnostics and the use of artificial intelligence tools, showing their superiority over classical statistics and encouraging the continuators. Some of the examples are listed below.

The introduction of newly discovered proteomic markers of early organ damage, such as the urinary exosomal WT-1 in diabetic nephropathy, is a promising new parameter that can detect patients before the clinical manifestation of organ damage, including increased UPCR, occurs [75,76].

Chronic kidney disease is a multisystem dysfunction, with cardiovascular complications leading to life-threatening conditions. Combining clinical parameters and patient/family history in a prognostic model with proteomic data offers the potential for tools to classify patients into risk groups for cardiovascular events, including sudden cardiac death. According to current knowledge, patients with chronic dialysis are at increased risk of cardiac events. Connective tissue growth factors (CTGF) and NT-proBNP play a significant diagnostic role, improving the ability to identify patients at risk for cardiovascular mortality (AUC 0.883) or sudden cardiac death (AUC 0.877) during a 2-year follow-up period. Although nonspecific, the CTGF and NT-proBNP biomarkers reflect the level of myocardial fibrosis and myocardial overload, respectively [77].

Idiopathic nephrotic syndrome is the most common primary glomerulopathy among children. Its beginning at a young age carries the burden of steroid-based and long-term immunosuppression. In a study by Yagin et al., predictive models were developed to identify steroid-dependent and steroid-resistant nephrotic syndrome in children based on a metabolic profile, taking into account many parameters, in particular serum glucose, creatinine, glycerate, carnitine, betaine [78]. The best model differentiated the two conditions with an accuracy of 0.87 and an AUC of 0.92. Indeed, in this case, artificial intelligence addressed a fundamental issue of nephrology in its entirety.

IgA nephropathy, the most common primary form of mesangial glomerulonephritis in adults, plays a significant role in the development of chronic kidney disease. There are promising biochemical marker studies that, when combined with artificial intelligence tools, enable identification of patients at risk of disease progression before glomerular structure is damaged [79].

Contrast-induced nephropathy constitutes a niche of nephrological conditions, yet the increasing demand for imaging diagnostics urges early identification of risk groups. Lee et al. and Gonzales et al. point to the potential for developing models predicting contrast-induced nephropathy, based on classic markers of renal function and multiple markers of early damage [80,81].

Kidney transplant patients are exposed to the adverse effects of the immunosuppressant tacrolimus, which, on one hand, protects the transplanted organ from rejection and, on the other, triggers nephrotoxicity, which becomes irreversible at some point. The potential targets for drugs, that can inhibit fibrosis induced by chronic tacrolimus administration, were identified. These include PRMT1, the presence of which triggers increased STAT3 levels and, consequently, increased expression of β6 subunits in the αVβ6 integrin, which is known to be elevated in chronic kidney disease [82].

The most common histological type of kidney cancer, accounting for 70% to 90% of all cases, is clear cell renal cell carcinoma (ccRCC), characterized by significant heterogeneity. Modeling based on upregulation and downregulation of genes selected through statistical analysis allows patient stratification for survival and therapeutic targeting [83].

The authors of all above-mentioned studies point out that the multitude of parameters allows for personalized medicine, tailored to every patient—those suffering from acute dysfunction, either developing chronic disease with comorbidities, or undergoing medical procedures. Therefore, the use of AI-driven analyses may serve for holistic purposes including diagnosis, treatment, and prophylaxis.

## 10. Back to the Future of Nephrology with New Markers

The future of artificial intelligence applications in medicine lies in network models, analyzing interactions between entities like proteins, genes, cells, or drugs, and leading to personalized management. Although the data on building such models in nephrology is scarce, the examples listed below, concerning other specialties, provide the area for future investigation and show the most effective tools.

Correctly registered molecular structures can be analyzed using artificial neural network models to discover the most effective catalytic reaction systems. The models then analyze the individual functional structures of the drug and target. Deep learning analysis of molecular models provides information that can be used to saturate molecular simulations with data on critical molecule–drug interactions. Such models achieve AUC above 0.9 [84]. Previous research highlights the importance of high-quality data provided for analysis [85].

The analysis of drug–target point interactions may enable the foundation of modern personalized medicine, focused on effective treatment with the most appropriate medication. The work of Mess et al. shows that models based on RFCs and XGBoost classifiers are effective. These models were tasked with predicting the target point for a selected drug in metastatic cancer (AUCs of 0.93 and 0.94, respectively), and in Parkinson’s disease (AUCs of 0.91 and 0.92, respectively) [86].

Artificial intelligence models are able to analyze information on the overexpression of numerous genes, representing the complexity of biochemical processes and numerous intercellular interactions involving proteins, cytokines, and chemokines, in the form of a digital, scalable model. Models examining intercellular interactions at the expression level of selected gene packages offer the potential to classify SARS-CoV-2 disease into severity categories. The deep learning network is able to identify gene expression patterns typical of immune diseases that correlate with the clinical severity of SARS-CoV-2 infection [87]. In short, the model established potential pathways of gene interactions based on measured gene expression. Machine learning models, despite their clearly simpler structure compared to deep learning, are not inferior in terms of predictive ability in the case of COVID-19. Classically determined biochemical parameters were of good predictive value, but extending the data with metabolomics significantly improved the precision of the prediction of SARS-CoV-2 infection severity. Not surprisingly, the best model was that of RFC. The kNN classifier proved to be of comparable quality, because they both achieved an AUC of 0.92 [88].

Deep learning identifies intercellular interactions at the molecular level that defy classical statistical analysis. Kim et al. were able to analyze over 36,000 genes in nearly 500,000 non-small cell lung cancer cells and over 700,000 colorectal cancer cells using this method [89]. On average, both models performed extremely well, achieving performance, as measured by accuracy and AUC, of 0.991 (AUC of 1.0) and 0.952 (AUC of 0.99), respectively [89].

Last, but not least, nonspecific markers, when combined with others, can provide significant prognostic potential. For example, the combination of interleukin 8- and 10-, TNFα, Brain-derived neurotrophic factor (BDNF), Nerve Growth Factor (NGF), and the chemokine CXCL10, arranged in a decision tree model, quite accurately differentiates treatment-resistant lower urinary tract dysfunction (LUTD) [90]. Therefore, it is clear that properly collected and processed data can yield promising results in AI-based research in the future.

## 11. Ethical Aspects of AI in Nephrology

Ethical considerations regarding AI in medicine focus primarily on responsibility for the results and outcomes of achieved diagnostic goals. In this regard, the degree of reliance on the chosen method and personal responsibility for the accuracy of the classification are crucial. The allocation of benefits from the achieved results remains an ethical issue, especially in the field of scientific work. Among the important technical issues, there are the reliability and flexibility of solutions [91,92].

The cited literature presents a developing picture of new tools that can support the care of patients with chronic kidney disease, end-stage renal failure, including dialysis patients and patients after kidney transplantation. It is important to critically assess the results achieved, so that they serve the patient’s well-being and have a positive impact on science.

## 12. Conclusions

Various machine learning techniques are characterized by differential performance in relation to the input data. The main requirement is the completeness of the data used to generate the model. Moreover, capabilities of a model usually increase with its complexity, although under certain conditions, a simpler model may perform better. Simpler models show some insensitivity to missing data or outlier data.

Further AI applications in nephrology are highly expected. In order to make the most of AI potential, basic rules of its efficient implementation, summarized in this review, have to be taken into account.

## Figures and Tables

**Table 1:** A compilation of various machine learning applications in proteomics and molecular sciences. ALB—albumin, AFM—afamin, ANXA7—annexin A7, APOD—Apolipoprotein D, C9—Complement component 9, SERPINA5—Protein C inhibitor, VPS4A—vacuolar protein sorting-associated protein 4A, CP—Ceruloplasmin, TF—transcription factor.

Authors	AI Method	Input Variables	Target Point	Performance
Yuan et al. [10]	LASSO logistic regression, RFC, SVM-RFE	Genes ARID4B, EOMES, KCNJ3, LIF and STAT1	Kidney fibrosis	AUC of 0.923
Kha et al. [20]	RFC, XGBoost, ET, LGBM, MLP	18 values derived from molecular computational methods	Possible drug–food constituent interactions (DFIs)	Accuracy of 96.75% for XGBoost
Massy et al. [11]	Clustering and Regularized Cox Regression	Set of 90 urinary peptides	Kidney failure	AUC of 0.83
Reznichenko et al. [5]	Self-Organizing Maps unsupervised ANN ML algorithm	A set of upregulated and downregulated genes associated with faster progression of chronic kidney disease (CKD)	CKD reclasification	AUC of 0.825
McCallion et al. [31]	SVM	CKAP4, PTX3, IGFBP2, OPN	Senescence in AKI and CKD vs. comorbidities	AUC of 0.98 for CKAP4
Schork et al. [6]	LASSO regression	Among 112 peptides CKD273, HF2, and CAD238 were statistically significant	Clustering into groups at risk of developing diabetes and diabetes complications	AUC of 0.868 with 95% CI 0.755–0.981
Deo et al. [12]	Elastic Net Regression	Set of 16 proteins	Secondary cardiovascular events	AUC of 0.77–0.80
Fan et al. [23]	Logistic regression	Sets of 2, 3 and 4 proteins: ALB + AFM, ANXA7 + APOD + C9, SERPINA5 + VPS4A + CP + TF	DKD vs. uncomplicated diabetic patients and DKD3 vs. DKD4, progression to DKD	AUC of 0.928, AUC of 0.949, AUC of 0.952, respectively
Kononikhin et al. [24]	Logistic regression, k-NN, SVM	Set of GPX3, PLMN, and A1AT or SHBG	Mild vs. severe glomerulopathies	AUC of 0.99
Glazyrin et al. [32]	KNeighbors (kNN), logistic regression, support vector machine (SVM), and decision tree	Set of biochemistry parameters, clusters	Various groups of chronic kidney disease of renal and postrenal or systemic etiology (prerenal)	Accuracy of 96.3% for glomerulonephritris and 96.4% diabetic nephropaty

**Table 2:** General characteristics of machine learning methods applications, requirements, leading advantages and disadvantages.

			Method	Application	Requirements	Advantages	Disadvantages
Artificial intelligence/ Machine learning			Support Vector Machine	Classification and regression of tabular data [27,30,34]	Complete data	Simplicity	Insufficient in more complex tasks, possible low performance on large sets
			Random Forest Classifier	Classification [34] and regression of tabular data Feature importation [50,53]	Complete data, partially insensitive to missing data	Simplicity, insensitivity to irrelevant variables, partial resistance to outliers	May be insufficient in more complex tasks
			XGBoost	Classification and regression of tabular data [26,27,28,29,34]	Complete data	Simplicity	
			kNN	Data imputation [35] regression, classification [32,34]	Empty cells for imputation	Simplicity	Possible low performance on large sets with many variables. Sensitive to missing and outlier data. Except for data imputation, rather limited use
	Artificial Neural Network		Multilayer Perceptron	Classification and regression of tabular data [34,41,42]	Necessary data scaling, standardization, improved performance	Can learn non-linear relationships Can learn up to date through additional data	Hyperparameter tuning required Sensitive to scaling of input data
		Deep learning	Convolutional Neural Network	Classification [38,43,48], deviation detection [43], segmentation [45,46,47,48]	Mainly imaging, histopathological, radiological data, etc.	Complexity enabling deep image analysis. Possibility to upload large amounts of data. Application of a once trained network in various approaches	Complexity, risk of overfitting to too few input data due to the multitude of parameters inside the model. Requirement of a sufficiently large number of class representatives
			U-Net [45,46,47]				
			ResNet18 [38] ResNet50 [38] ResNet101 [38,48]				

## Data Availability

No new data was created or analyzed in this study. Data sharing is not applicable to this article.

## Abbreviations

The following abbreviations are used in this manuscript:

A1AT	Alpha-1 antitrypsin
ADPKD	Autosomal dominant polycystic kidneys disease
AFM	Afamin
AI	Artificial intelligence
AKI	Acute kidney injury
AlexNet	Alex Krizhevsky Network
ANN	Artificial neural network
ANXA7	Annexin A7
APOD	Apolipoprotein D
ARID4B	AT-rich interactive domain-containing protein 4B
AUC	Area under curve
BDNF	Brain-derived neurotrophic factor
BMI	Body mass index
C9	Complement component 9
CAD238	Coronary Artery Disease 238
CARTs	Classification and regression trees
ccRCC	Clear cell renal carcinoma
CKAP4	Cytoskeleton-associated protein 4
CKD273	Chronic kidney disease 273
CNN	Convolutional neural network
CP	Ceruloplasmin
CTGF	Connective tissue growth factors
CXCL10	C-X-C motif chemokine ligand 10
DGF	Delayed graft function
DKD	Diabetic kidney disease
DoS	Difference of slope
eGFR	Estimated Gloimerular Filtration Rate
EOMES	Eomesodermin
EPTS	Estimated post-transplant survival
ESAs	Erythropoiesis-stimulating agents
ESKD	End-stage kidney disease
ET	Extra trees
FN	False negative
FP	False positive
FPR	False positive rate
GIRK-1	G protein-activated inward rectifier potassium channel 1
GPX3	Glutathione Peroxidase 3
Hb	Hemoglobin
HF2	Heart failure 2
HR	Heart rate
ICU	Intensive Care Unit
IGFBP2	Insulin-like Growth Factor-Binding Protein 2
KDPI	Kidney Donor Profile Index
KDRI	Kidney Donor Risk Index
kNN	K-nearest neighbors classifier
LASSO	Least Absolute Shrinkage and Selection Operator
LGBM	Light Gradient-Boosting Machine
LIF	Leukemia inhibitory factor
LOOCV	Leave-one-out cross-validation
LUTD	Lower urinary tract dysfunction
MCC	Matthews correlation coefficient
MLP	Multilayer perceptron
NGF	Nerve Growth Factor
OPN	Osteopontin
PLMN	Plasminogen
PRMT1	Protein Arginine Methyltransferase 1
PTX3	Pentraxin-3
ResNet	Residual Network
RFC	Random Forest Classifier
RFCs	Random Forest Classifiers
RI	Resistive index
ROC	Receiver-operator curve
sAlb	Serum albumin
SHBG	Sex hormone binding globulin
STAT1	Signal transducer and activator of transcription 1
STAT3	Signal transducer and activator of transcription 3
SVM	Support vector machine
SVM-RFE	Support vector machine recursive feature elimination
SWE	Shear wave elastography
TF	Transcription factor
TN	True negative
TP	True positive
TPR	True positive rate
UACR	Urinary albumin creatinine ratio
UCI	University of California Irvine
U-Net	U-shaped architecture Network
VPS4A	Vacuolar protein sorting-associated protein 4A
XGBoost	eXtreme Gradient Boosting
